# Cardiovascular Control during Exercise in Type 2 Diabetes Mellitus

**DOI:** 10.1155/2015/654204

**Published:** 2015-03-30

**Authors:** Simon Green, Mikel Egaña, J. Chris Baldi, Regis Lamberts, Judith G. Regensteiner

**Affiliations:** ^1^School of Science and Health, University of Western Sydney, Sydney, NSW 2751, Australia; ^2^Neuroscience Research Australia, Sydney, NSW 2751, Australia; ^3^Department of Physiology, School of Medicine, Trinity College Dublin, Dublin 1, Ireland; ^4^Department of Medicine, University of Otago, Dunedin, Otago 9054, New Zealand; ^5^Department of Physiology-HeartOtago, University of Otago, Dunedin, Otago 9054, New Zealand; ^6^Division of General Internal Medicine, Center for Women's Health Research, Department of Medicine, School of Medicine, University of Colorado, Denver, CO 80210, USA

## Abstract

Controlled studies of male and female subjects with type 2 diabetes mellitus (DM) of short duration (~3–5 years) show that DM reduces peak $\dot{\text{V}}{\text{O}}_{2}$ (L·min^−1^ and mL·kg^−1^·min^−1^) by an average of 12–15% and induces a greater slowing of the dynamic response of pulmonary $\dot{\text{V}}{\text{O}}_{2}$ during submaximal exercise. These effects occur in individuals less than 60 years of age but are reduced or absent in older males and are consistently associated with significant increases in the exercise pressor response despite normal resting blood pressure. This exaggerated pressor response, evidence of exertional hypertension in DM, is manifest during moderate submaximal exercise and coincides with a more constrained vasodilation in contracting muscles. Maximum vasodilation during contractions involving single muscle groups is reduced by DM, and the dynamic response of vasodilation during submaximal contractions is slowed. Such vascular constraint most likely contributes to exertional hypertension, impairs dynamic and peak $\dot{\text{V}}{\text{O}}_{2}$ responses, and reduces exercise tolerance. There is a need to establish the effect of DM on dynamic aspects of vascular control in skeletal muscle during whole-body exercise and to clarify contributions of altered cardiovascular control and increased arterial stiffness to exertional hypertension.

## 1. Introduction

Type 2 diabetes mellitus (DM) results in a loss of cardiorespiratory fitness and exercise tolerance and accentuates the blood pressure response during exercise. Understanding the physiological changes underlying these altered exercise responses is important to the design of therapeutic interventions aimed at helping individuals with DM increase their physical activity levels and fitness and reduce their cardiovascular risk [1, 2]. In recent years, reviews have been written about exercise and cardiovascular function in DM [1, 2]. However, none of these reviews interpreted the available evidence within a context of cardiovascular control during exercise, and doing so provides a different insight into mechanisms underlying exercise intolerance and associated cardiovascular consequences of DM.

In this review, we introduce a simplified conceptual model of cardiovascular control during exercise before reviewing the effects of diabetes on time- and intensity-dependent features of cardiovascular control during exercise. In doing so, we also explore the interactions between cardiovascular responses and oxygen uptake ($\dot{\text{V}}{\text{O}}_{2}$) given the physiological relationships between them and the importance of $\dot{\text{V}}{\text{O}}_{2}$ to exercise tolerance, cardiorespiratory fitness, and mortality.

## 2. Cardiovascular Control during Exercise

The cardiovascular system is “designed” to perfuse cells of all tissues [3]. Each tissue has control over its own perfusion, or blood flow, but this “local” level of control depends upon a sufficiently high level of arterial pressure maintained by a “central control mechanism” [4, 5]. Local* control* of blood flow occurs within system-wide* regulation* of systemic arterial blood pressure. From a systems control perspective, the central regulation of blood pressure is perhaps the most important feature of the cardiovascular system because it enables the local control of blood flow to occur.

**Figure 1:**
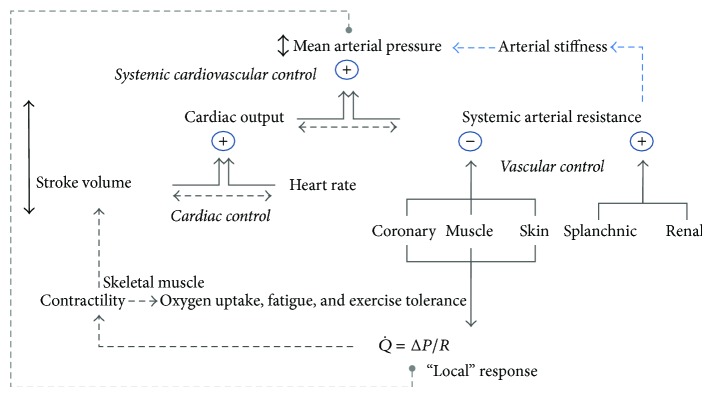
A simple conceptual model of cardiovascular control during exercise with links to oxygen uptake, fatigue, and exercise tolerance. Definitions of terms and description of most interrelationships between variables are described in the main text. The length of bidirectional arrows indicates the relative ranges of variables: the* regulated* variable, mean arterial pressure, varies through a smaller range than* controlled* variables such as cardiac output and systemic arterial resistance. Dashed bidirectional arrows indicate interactions between two variables. Arterial stiffness is not considered to be a part of a system of cardiovascular regulation and control, but it is influenced by this system (systemic arterial resistance) and also exerts an independent influence on mean arterial pressure.

A simple, conceptual model of cardiovascular control during dynamic exercise is shown in Figure 1. In this model, the regulation of blood pressure during exercise is reflected in the relatively small change in mean arterial pressure (MAP) that occurs across a wide range of work rates [6]. This regulation of MAP is effected through the control of several responses involving the heart (“cardiac control”), blood vessels (“vascular control”), and their combined effects (“systemic cardiovascular control”). Systemic cardiovascular control reflects the interactions between MAP, cardiac output (CO), and systemic vascular resistance (SVR). During exercise, cardiac output and SVR can be adjusted across a much larger response range compared with MAP. Cardiac output is controlled through adjustments to heart rate (HR) and stroke volume (SV) and these three variables constitute aspects of cardiac control. Systemic vascular resistance is controlled by vasodilation in some tissues (skeletal muscles, heart, and skin) and vasoconstriction in others (splanchnic region and kidneys), with the net effect being a decline in SVR. The fall in SVR during exercise is immediate, driven largely by “rapid vasodilation” in contracting muscles, which is counterbalanced by a slightly delayed rise in CO so that MAP falls transiently and then rises to a level above the resting value and proportional to the work rate [6–8]. In this way, MAP is effectively regulated through the controlled increase of cardiac output in the presence of a controlled reduction in SVR.

Blood flow through a tissue ($\dot{Q}$) is proportional to the perfusion pressure (Δ*P*) and inversely proportional to the total resistance acting across the tissue vascular bed (*R*) ($\dot{Q}=\Delta P/R$). Effects of changes in coronary and skeletal muscle blood flow on contractility, SV, and motor output (fatigue, exercise tolerance) are also illustrated, as are interactions between controlled variables (e.g., HR-SV interactions). For simplicity, the model excludes potentially important influences of venous capacitance, compliance, and resistances on perfusion pressure and CO [5] and it does not, for example, distinguish between respiratory and locomotor skeletal muscles and interactive or “competitive” effects between them or other vascular beds, such as in the skin [9, 10].

## 3. Effect of Type 2 Diabetes Mellitus on Peak and Dynamic Responses of Oxygen Uptake

Given the close association between cardiovascular function and pulmonary $\dot{\text{V}}{\text{O}}_{2}$, the maximum and dynamic responses of pulmonary $\dot{\text{V}}{\text{O}}_{2}$ during exercise provide insight into the scope and speed of cardiovascular control and whether or not they are affected in DM. Maximum or “peak” $\dot{\text{V}}{\text{O}}_{2}$ responses are generally measured at the end of a maximum graded exercise test, and in this review we refer to the “peak” value as the highest value measured during this type of test. Dynamic aspects of $\dot{\text{V}}{\text{O}}_{2}$ are usually assessed during submaximal exercise at a fixed workload low enough to ensure steady-state $\dot{\text{V}}{\text{O}}_{2}$ is achieved within 2-3 minutes. The $\dot{\text{V}}{\text{O}}_{2}$ response during submaximal exercise consists of at least two phases and the time constant of the second phase of pulmonary oxygen uptake ($\dot{\text{V}}{\text{O}}_{2}\tau$) reflects an important dynamic aspect of the overall response.

Evidence related to the effects of DM on these peak and dynamic $\dot{\text{V}}{\text{O}}_{2}$ responses in men and women, as well as adolescents, is shown in Table 1. The diabetes of these subjects was “uncomplicated” in that they did not have overt cardiovascular dysfunction at rest and, also, the duration of diabetes was limited to 4–6 years. Values of peak $\dot{\text{V}}{\text{O}}_{2}$ have been expressed in L·min^−1^ rather than normalised to body weight (e.g., mL·kg^−1^·min^−1^) because of the bias introduced by consistently higher body weights (and BMI) of DM compared with control subjects. Most data in Table 1 pertains to middle-aged subjects (~40–60 y) in whom DM consistently reduced peak $\dot{\text{V}}{\text{O}}_{2}$ and increased $\dot{\text{V}}{\text{O}}_{2}\tau$ in males and females. The effect of DM was greater for $\dot{\text{V}}{\text{O}}_{2}\tau$ (34–36%) than peak $\dot{\text{V}}{\text{O}}_{2}$ (12–15%), suggesting a greater level of impairment of the dynamic compared with maximum response. Although exercise tolerance data (i.e., peak power or exercise time) are not shown in Table 1, the magnitude of effect of DM on exercise tolerance (~10–20%) is similar to its effects on peak $\dot{\text{V}}{\text{O}}_{2}$. More limited data from older subjects (>60 y, males) suggest a similar effect of DM on peak $\dot{\text{V}}{\text{O}}_{2}$ to effects observed in younger men but show that $\dot{\text{V}}{\text{O}}_{2}\tau$ is not affected by DM. This differential effect of DM on peak $\dot{\text{V}}{\text{O}}_{2}$ and $\dot{\text{V}}{\text{O}}_{2}\tau$ might be attributed to a greater effect of ageing on $\dot{\text{V}}{\text{O}}_{2}\tau$ in older individuals that effectively masks the effect of DM on $\dot{\text{V}}{\text{O}}_{2}\tau$ seen in younger individuals [11].

## 4. Effect of Diabetes on Cardiovascular Control during Maximum Graded Exercise

### 4.1. Blood Pressure

The regulation of MAP during exercise is central to cardiovascular control (Figure 1) and alterations in how it is regulated might be clinically important. Measurements of MAP* during* maximum graded exercise provide important insight into how diabetes affects the regulation of blood pressure as the workload and cardiovascular strain are progressively increased towards maximum levels.

Two studies assessed arterial blood pressure (manual sphygmomanometry) at rest and during the final stage of maximum graded exercise in adults with DM (BMI ≈ 32 kg·m^−2^) and controls (BMI ≈ 26 kg·m^−2^) [12, 13]. Differences between studies included sample size (*n* = 20 versus 146), age (~40 versus 54 y), sex (premenopausal women versus older men and women), and exercise mode (treadmill versus cycle ergometer). Despite these differences, resting MAP was similar between DM and controls (2 mmHg difference). By contrast, peak MAP was 10–13 mmHg (*P* < 0.05) higher in DM groups which resulted in a 50% greater pressor response in DM (~29-30 mmHg) than controls (~19–21 mmHg). This greater pressor response was due to a greater increase in diastolic (9 versus 5 mmHg) and systolic (72 versus 61 mmHg) blood pressures [13]. Similar effects of DM on the maximum exercise pressor response were recently observed in adolescent male and female subjects [14].

### 4.2. Cardiac Output (CO) and Systemic Arterial Resistance (SAR)

The effect of DM on the maximum exercise pressor response reflects an altered balance between the maximum rise and fall in CO and SAR (Figure 1). Three studies shed light on this [12, 15, 16] and showed that resting CO is normal in DM but that the peak response is blunted.

The first of these studies by Roy et al. [16] showed that patients with more advanced DM and cardiac autonomic dysfunction had 15–30% lower peak CO than controls. However, interpretation of this study is undermined by inadequate description of the CO technique, lack of data concerning $\dot{\text{V}}{\text{O}}_{2}$ and power output, as well as substantial differences in resting cardiac output which bias the way in which data were presented (i.e., data were normalised to resting values). More recent studies of males and females with uncomplicated DM show more modest reductions (~5–10%) of peak CO, where CO was assessed using the CO_2_-rebreathing technique, thermodilution (pulmonary artery), and the direct Fick method [12, 15].

Systemic arterial resistance is estimated from MAP and CO measurements. At rest, SAR is normal in male and female subjects with uncomplicated DM [11, 12, 20]. During maximum graded exercise, the fall in SAR, based on direct Fick measurements of CO, was slightly lower in DM (53%) than controls (60%) [12]. Although this effect was not significant, it is of a magnitude (~10%) that helps explain the significant effect of DM on the exercise pressor response.

### 4.3. Heart Rate and Stroke Volume

The blunted rise in cardiac output during maximum graded exercise in DM must be due to a smaller increase in heart rate and/or stroke volume, and questions arise as to whether this is due to alterations in resting and/or peak responses.

At rest, HR is similar [12, 26] or up to 10% higher in uncomplicated DM [13, 27] and SV is comparatively similar [12] or slightly smaller by up to 10% [27] in DM. Given that resting cardiac output is not affected by DM [11, 12, 19, 20], these data suggest that the relative contributions of HR and SV to cardiac output can be altered in diabetes, although this is not always observed. When a reduction in resting SV has been observed, it has been associated with impairments of systolic function (e.g., low peak ejection rate) and diastolic function (e.g., slowed left ventricular filling) and a smaller circulating blood volume [27]. This latter observation is intriguing and, given the importance of blood volume to cardiovascular function during exercise, warrants further investigation.

More consistent evidence pertains to the effect of DM on peak HR (Table 1) with an average reduction based on all studies of 2.5–3.1% (Table 1). This, combined with higher resting HR, suggests that the scope for raising heart rate during maximum graded exercise is mildly reduced by DM. Data related to peak SV are much more limited and direct measurements of SV during the final stage of maximum graded exercise suggest a reduction in peak SV of 4% (Fick method) or 10% (thermodilution) [12]. Collectively, these data suggest that peak responses of HR and SV are mildly blunted in DM and that they contribute to reductions in peak CO and $\dot{\text{V}}{\text{O}}_{2}$.

### 4.4. Vascular Control

The DM-induced dampening of the fall in systemic arterial resistance, or rise in systemic vascular conductance, during maximum graded exercise could be due to effects in one or more vascular beds involved in this response (Figure 1). There appears to be no evidence pertaining to these effects during whole-body exercise. However, the dominant influence of vasodilation in contracting skeletal muscle on this systemic response [6] raises the possibility that DM impairs vascular control in contracting muscle. Studies of isolated limb exercise shed light on this effect and insight into vascular control can be inferred from measurements of limb blood flow and, particularly, limb vascular conductance (limb blood flow/MAP).

Kiely et al. [28] studied calf blood flow and vascular conductance responses during maximum graded calf exercise in middle-aged males and females with uncomplicated DM (*n* = 44) and controls (*n* = 35). Performance on this test (i.e., peak force normalised to MVC) was reduced by 13–16% in DM men and women, similar to the effect of DM observed for whole-body exercise. Resting and peak calf blood flow and vascular conductance were lower in subjects with DM, and the maximum rise in leg vascular conductance (peak − rest) was also lower in males (14%) and females with DM (26%). Although MAP increased by 11–15 mmHg during calf exercise, it was not affected by DM.

Thus, consistent with the effect of DM on the maximum rise in systemic vascular conductance (or decline in SAR) during whole-body exercise, DM appears to blunt the maximum response of vasodilation during exercise isolated to a small group of lower limb muscles. Potential mechanisms underlying this response are discussed in a later section.

## 5. Effect of Type 2 Diabetes on Steady-State Cardiovascular Responses during Submaximal Exercise

The effects of DM on peak cardiovascular responses during maximum graded exercise raise questions about whether or not these effects begin to appear at lower workloads. Steady-state measurements during submaximal exercise might shed light on this.

### 5.1. MAP

Kingwell et al. [29] measured femoral arterial blood pressure at rest and during 25 minutes of supine cycle ergometer exercise at 60% peak $\dot{\text{V}}{\text{O}}_{2}$ in nine DM men and nine healthy men. Resting pressures were not different between groups, but during the final 15 minutes of exercise the systolic, diastolic, and mean pressures were higher in DM (155, 79, and 104 mmHg) than controls (131, 68, and 89 mmHg). In two other studies involving males and females, O'Connor et al. showed that the rise in MAP (manual sphygmomanometry) by the fourth minute of moderate exercise (80% ventilatory threshold) was 5–10 mmHg higher in middle-aged and older subjects with DM [11, 20]. These studies demonstrate that DM evokes an increase in the pressor response during moderate exercise that is of a similar magnitude to that observed for the peak response during maximum graded exercise. This suggests that the maximum effect of DM on the pressor response is already evident at moderate workloads.

### 5.2. Cardiac Output, Heart Rate, and Stroke Volume

Several studies assessed CO responses during submaximal, steady-state exercise (~60–70% peak $\dot{\text{V}}{\text{O}}_{2}$) using gas-rebreathing techniques in middle-aged and older males and females with DM and age- and sex-matched controls [11, 15, 19, 20]. When the exercise-induced increase in CO is normalised to power output (i.e., gain of CO), data from these studies show a consistent lack of effect of DM on this response. Moreover, the gain of the HR or SV responses was similar between DM and controls [11], suggesting that their contributions to the increase in CO were not affected by DM. By contrast, recent evidence of these responses in adolescents suggests that the increases in SV and CO were blunted in DM [14]. Why this outcome differs from studies of adults is not clear, but we note that the resting blood pressure and SAR were ~10% higher than normal in the adolescents with DM, whereas this has not usually been observed in adults.

### 5.3. Systemic Arterial Resistance (SAR)

Simultaneous measurements of MAP and CO soon after the achievement of steady-state (*t* = 4 min) during moderate exercise show that the fall in SAR [20] and rise in systemic vascular conductance [11] were blunted in middle-aged males and females with DM. Such effects cannot be attributed to the lower power outputs generated by DM subjects, because the gain of the response of systemic vascular conductance was 50% lower in DM than controls at the same relative intensity [11]. This indicates that the underlying vasodilation in contracting muscles was more constrained in DM, suggesting impaired vascular control in DM.

### 5.4. Limb Blood Flow and Vascular Conductance

Studies of limb blood flow and vascular conductance (or resistance) during exercise include those which have assessed it during cycle ergometer exercise [29, 30] and isolated contractions involving a single limb [21, 28, 31].

Kingwell et al. [29] measured blood flow in the right femoral vein (thermodilution) during 25 minutes of supine cycle ergometer exercise (91–97 W, 60% peak $\dot{\text{V}}{\text{O}}_{2}$) in diabetic and healthy men. Resting limb blood flow was not different between these groups, whereas exercise responses (*t* = 10–25 min) were 25% lower in DM. Arterial blood pressures were higher during exercise (but not rest) in DM and, consequently, the reported mean value of leg vascular resistance during exercise was 52% higher than control subjects (42.5 versus 27.9 mmHg·min^−1^·L^−1^). These data are consistent with the effects of DM on SAR during moderate exercise and suggest that the simultaneous decrease in vascular resistance in contracting muscles is blunted in DM.

However, Kingwell et al. pointed out that this finding differed from an earlier finding of a lack of effect of DM on limb blood flow (thermodilution) under very similar exercise conditions [32], but in the upright position, and attributed this effect to differences in fitness levels between the study cohorts. Interpretation of their data should also consider a companion study that involved the same DM and control subjects [30] and provided Fick estimates of “leg” (limb) $\dot{\text{V}}{\text{O}}_{2}$. Limb $\dot{\text{V}}{\text{O}}_{2}$ was 15% lower in DM (0.408 L·min^−1^) than controls (0.477 L·min^−1^) despite the similar power output and pulmonary $\dot{\text{V}}{\text{O}}_{2}$, suggesting that the lower blood flow in DM is linked to a lower oxygen consumption in those muscles (thigh and leg) “sampled” by thermodilution. In this respect, the powerful hip extensors might contribute more to power output and $\dot{\text{V}}{\text{O}}_{2}$ in DM than controls, and blood flow to these muscles is* not* assessed by thermodilution catheters placed in the femoral vein.

Problems related to the control and measurement of a submaximal workload in contracting muscles can be reduced when exercise is limited to single muscle groups. MRI measurements of CO and femoral arterial blood flow were made in eight men with DM and 11 healthy controls while they performed “low-intensity” knee extensor exercise that involved repeated lifting of a 1.5 kg weight at 1 Hz [21]. CO and femoral arterial blood flow increased significantly in both groups, but the exercise value for arterial blood flow (and not CO) was significantly lower in DM. This prompted the suggestion that the muscle hyperaemic response was more constrained in DM and unrelated to the CO response [21]. However, calculations based on their data show that the exercise-induced* change* in these responses was lower in DM for femoral arterial flow (28%) and CO (67%). In the absence of determinations of workload (power output) and the tendency (*P* = 0.20) for diabetic men to be shorter than control men, there remains the possibility that the power output of this exercise was relatively less for DM and it helps explain their lower muscle hyperaemic and CO responses.

A more recent study of 79 middle-aged men and women [28] reports data on responses of vascular conductance in calf muscles during low-intensity (30% MVC), intermittent contractions of the plantar flexors. Workloads were carefully controlled and not different between DM and control subjects, and calf blood flow was measured using venous occlusion plethysmography [33–35]. Although resting calf blood flow and vascular conductance were lower in DM than controls, exercise-induced changes in leg vascular conductance were not significantly lower in women (2.46 versus 3.27 mL·min^−1^·mmHg^−1^) or men (2.58 versus 2.24 mL·min^−1^·mmHg^−1^).

These three studies provide mixed evidence about the effect of DM on the muscle hyperaemic response under submaximal, steady-state conditions, and further research is required to clarify this.

## 6. Effect of Type 2 Diabetes Mellitus on Dynamic $\dot{\text{V}}{\text{O}}_{2}$ and Cardiovascular Responses during Submaximal Exercise

Steady-state measurements provide little insight into how quickly $\dot{\text{V}}{\text{O}}_{2}$ and the cardiovascular system respond at the onset of exercise. Slow adjustments in muscle blood flow and $\dot{\text{V}}{\text{O}}_{2}$ can result in an altered internal metabolic state (e.g., increased [P_i_], [ADP], and [H^+^]), membrane potential (e.g., increased interstitial [K^+^] and intracellular [Na^+^]), and contractility of contracting skeletal myocytes. These effects may result in increased muscle fatigue and blood pressure during submaximal exercise, as well as a reduction in exercise tolerance.

### 6.1. Oxygen Uptake

Table 1 provides compelling evidence that DM increases $\dot{\text{V}}{\text{O}}_{2}\tau$ in men and women below the age of ~60 years. This demonstrates that the rate at which the “primary” phase of pulmonary $\dot{\text{V}}{\text{O}}_{2}$ increases towards its steady-state level is consistently slowed in these subjects with DM. This phase begins ~15–40 s after exercise onset and is linked closely to oxygen consumption by contracting muscles [36]. Thus, the DM-induced slowing of this primary phase implies impairment of the underlying control of O_2_ delivery to and/or utilisation of O_2_ by contracting muscles. The control of O_2_ delivery is dependent on cardiovascular control before and/or during this primary phase.

**Figure 2:**
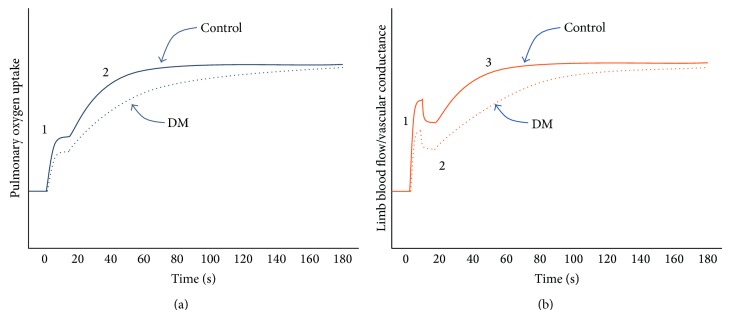
(a) Effect of type 2 diabetes mellitus (DM) on the dynamic response of pulmonary oxygen uptake during submaximal exercise below the ventilatory threshold. DM blunts the cardiodynamic phase (“1”) and slows the rise (increases the time constant) of the primary phase (“2”). Note that a third phase (“slow component”) is observed at higher intensities and can also be observed in a minority of subjects below the ventilatory threshold, but the effect of DM on this phase is not clear. (b) Effect of DM on the dynamic response of limb blood flow and vascular conductance during submaximal contractions (calf muscle). DM blunts the fast growth phase (“1”), has minimal effect on the rapid decay phase (“2”), and slows the rise (increases the time constant) of the slow growth phase (“3”). Note that a fourth phase, a slow decay, is observed in some subjects but it is not shown in this figure and the effect of DM on it is not clear.

However, it is worth noting that the dynamic response of $\dot{\text{V}}{\text{O}}_{2}$ is not comprehensively described by a single parameter (*τ*) of a single phase because pulmonary $\dot{\text{V}}{\text{O}}_{2}$ during submaximal exercise exhibits at least two phases, each of which can be described by more than one parameter (e.g., amplitude, time delay, and time constant) (Figure 2(a)). At the onset of exercise, pulmonary $\dot{\text{V}}{\text{O}}_{2}$ rises rapidly within the first 1-2 breaths as a result of the abrupt increase in pulmonary blood flow. Unfortunately, this “cardiodynamic” phase, which precedes the “primary” phase, is often excluded from analysis on the basis that it has little connection with metabolic responses in contracting muscles. The recent observation that the amplitude of this cardiodynamic phase is blunted in DM [11] suggests that it is also affected by diabetes. Given that the increase in pulmonary blood flow (CO) during this phase is coupled with an increased “venous return” that is linked temporally (and probably functionally) to the rapid vasodilation and hyperaemia in contracting muscles [35, 37], the DM-induced blunting of this phase points to alterations in dynamic responses of cardiac output and, perhaps more fundamentally, muscle blood flow.

### 6.2. Cardiac Output

In accordance with the Fick equation for $\dot{\text{V}}{\text{O}}_{2}$, the dynamic response of pulmonary $\dot{\text{V}}{\text{O}}_{2}$ is the product of the dynamic responses of cardiac output ($\dot{Q}$) and arterial-venous concentration difference for O_2_ ([a-vO_2_]) across the pulmonary circulation. Insight into the dynamics of these responses requires a temporal resolution of measurement similar to that provided through breath-by-breath analysis of pulmonary gas exchange (i.e., ~2–5 s) which, to our knowledge, has not yet been achieved. Nevertheless, three studies from the same laboratory provide insight into dynamic responses of CO and [a-vO_2_] using a more temporally limited technique for assessing CO (SF6 plus N_2_O-rebreathing) at 30 s and 240 s into cycle ergometer exercise combined with breath-by-breath measurement of pulmonary $\dot{\text{V}}{\text{O}}_{2}$ throughout exercise.

These studies involved middle-aged women [19], middle-aged men and women [20], and middle- and older-aged men [11]. The timing of the first measurement of CO (30 s) was justified on the basis that any contribution of CO to slowing of the $\dot{\text{V}}{\text{O}}_{2}$ response (first and second phase) should be apparent during this period and given that the second phase was assumed to begin at ~20 s into exercise. All three studies showed a significant increase in the time constant of the second phase of $\dot{\text{V}}{\text{O}}_{2}$ in middle-aged diabetic subjects compared with controls, but in all cases the initial rise (to 30 s) and final value (240 s) of CO normalised to power output were not lower in DM subjects. These observations suggest that the rate of rise in CO* per se* does not contribute to the blunted first phase or slowed second phase of pulmonary $\dot{\text{V}}{\text{O}}_{2}$. Moreover, DM did not affect the contributions of HR and SV to these changes in CO.

This normal dynamic response of CO in the presence of slowed pulmonary $\dot{\text{V}}{\text{O}}_{2}$ dynamics in DM implies an altered dynamic response of pulmonary [a-vO_2_]. This was looked at more carefully in a recent study [11] which showed a DM-induced increase in $\dot{\text{V}}{\text{O}}_{2}\tau$ in middle-aged men but not older men. Consistent with this age-dependent effect of DM was a 36%* greater* increase in pulmonary [a-vO_2_] by 30 s of exercise in middle-aged DM men and no difference in this response between older DM men and controls. That pulmonary [a-vO_2_] was calculated from $\dot{\text{V}}{\text{O}}_{2}$ and CO measurements rather than direct measurements of arterial and venous [O_2_] means we cannot be certain which of these concentrations is affected by DM. However, the most probable explanation relates to a lower mixed-venous [O_2_] and a slowed dynamic response of muscle blood flow (see below).

### 6.3. Mean Arterial Pressure and Systemic Vascular Resistance

In this abovementioned study [11] the gain of CO (L·min^−1^·W^−1^) during the first 30 s of exercise was similar between younger and older DM men and their age-matched controls. However, the gain in MAP (mmHg·W^−1^) was more than twice as great in middle-aged DM men than control subjects which meant that the calculated change in systemic vascular conductance based on changes in CO and MAP was significantly smaller in these diabetic men. This effect of DM was not observed in older men. This suggests that DM dampens the rate of increase in systemic vascular conductance during the initial 30 s of exercise in the presence of a normal response of CO, resulting in a more pronounced rise in arterial blood pressure soon after the onset of exercise.

### 6.4. Limb Blood Flow and Vascular Conductance

Does DM slow the dynamic response of vasodilation and rise in vascular conductance in contracting skeletal muscles?

Recent studies of forearm [37] and calf contractions [35, 38] have helped resolve the dynamic response characteristics of limb vascular conductance during submaximal exercise. For calf exercise, the basic structure of the muscle hyperaemic response consists of two growth phases and one decay phase, and sometimes a second decay phase is observed (Figure 2(b)). Slowing of the overall hyperaemic response is most likely due to slowing of one or both growth phases and two recent studies shed light on this.

In the first study involving female subjects [31], intermittent calf contractions were performed in an upright position, at a high intensity (70% MVC), and calf vascular conductance was described using a biphasic (growth only) function. The amplitude of the fast growth phase was 17% lower (not significant) in DM than controls, and the time constant of the slow growth phase of calf vascular conductance was three times higher in DM (*τ* = 66 versus 22 s). In a more recent study of middle-aged men and women (total *n* = 69), the dynamic response of calf vascular conductance was assessed during low-intensity (30% MVC) and high-intensity (70% MVC) contractions in the supine position, and this response was fitted to a more complex function which accounted for the presence of decay phases [28]. There was no effect of DM on any dynamic response characteristic of calf vascular conductance during low-intensity exercise, but for more intense contractions there was a significant reduction in the amplitude of the fast growth phase in DM men (21%) and women (49%) and a significant increase in the time constant of the slow growth phase in DM men (*τ* = 29 versus 14 s) and women (*τ* = 44 versus 18 s). In both of these studies, the MAP responses during exercise were unaffected by DM. These studies show that the control of vasodilation and muscle blood flow during more intense contractions of human lower limb muscles is impaired in DM and that fast and slow growth phases of the hyperaemic response are affected.

## 7. Mechanisms of Impaired Vascular Control in Diabetes

This review shows that diabetes slows the dynamic response and blunts the maximum response of vasodilation in contracting human skeletal muscle. How this effect occurs is not clear. There is an extensive literature on DM and its effects on the vasculature as it relates to, for example, endothelial dysfunction [39], skeletal muscle in the resting state [40], and the impact of exercise training [41]. However, there is a poorer understanding of the effects of DM on vascular control during exercise.

Physiological control of vasodilation in contracting skeletal muscle is complex and involves mechanisms of mechanical, neural, endothelial, metabolic, and humoral origin. In the context of DM, there has been a tendency to equate vascular dysfunction with “endothelial dysfunction” [1, 2, 29] supported by evidence that DM impairs endothelial-dependent vasodilation in* resting* human limbs [29, 42]. However, DM also impairs endothelial-independent vasodilation [43] in* resting* limbs and this points to the potential importance of vascular smooth muscle and other mechanisms contributing to impaired vascular control in contracting skeletal muscle.

The locus of control of vasodilation in skeletal muscle resides mainly within its “resistance” vessels consisting of feed arteries and arterioles. Insight into the effect of DM on this vascular network comes from studies of animal models of the disease and the use of techniques (intravital microscopy) to visualise this network during muscle contractions. Such studies reveal that diabetes and prediabetes increase the vascular responsiveness to vasoconstrictors [44, 45] and blunt vasodilation during muscle contractions [45, 46]. This latter effect is attributed to either an increased vascular constraint, for example, by noradrenaline [47], and/or impaired coordination of ascending vasodilation from terminal to upstream arterioles and feed arteries involving intercellular coupling via gap junctions in endothelial* and* vascular smooth muscle cells [46, 48]. Such effects might contribute to the slowed dynamic response of muscle hyperaemia (vasodilation) in DM. In addition, animal models of diabetes studies reveal structural changes, such as arteriolar and capillary rarefaction, which on their own help explain the blunted maximal vasodilation in these models [49] and might help explain the effect of DM on maximal vasodilation during exercise in humans.

## 8. Synthesis: Cardiovascular Contributions to Impaired Pulmonary $\dot{\text{V}}{\text{O}}_{2}$, Exercise Intolerance, and Exertional Hypertension in Type 2 Diabetes Mellitus

There is compelling evidence that DM impairs the peak and dynamic responses of pulmonary $\dot{\text{V}}{\text{O}}_{2}$ (Table 1) in younger individuals with DM. Evidence of functional coupling between $\dot{\text{V}}{\text{O}}_{2}$ assessed “at the lung” and contracting skeletal muscles [36, 50, 51] implicates the latter in effects of diabetes on pulmonary $\dot{\text{V}}{\text{O}}_{2}$. Recent studies provide insight into the cardiovascular contributions to these effects, although interpretation of pulmonary measurements is complicated by variable delays, lags, and alterations in regional arterial and venous blood flows which affect the extent of temporal coupling between pulmonary and muscle $\dot{\text{V}}{\text{O}}_{2}$ responses and their underlying Fick determinants ($\dot{\text{V}}{\text{O}}_{2}=\dot{Q}\times [\text{a-v}{\text{O}}_{2}]$).

The effect of DM on peak $\dot{\text{V}}{\text{O}}_{2}$ (~12–15%) can be attributed to a reduced peak CO and pulmonary [a-vO_2_]. At least half of this effect on peak $\dot{\text{V}}{\text{O}}_{2}$ can probably be attributed to a reduced peak CO (~5–10%) [12, 15], heart rate (~3%), and stroke volume (~5%). The remaining effect appears to be linked to [a-vO_2_] which has been observed to be 20% lower in DM [15], although an effect has not always been observed [12]. Further studies are required to clarify the contributions of CO and [a-vO_2_] to the effect of DM on peak $\dot{\text{V}}{\text{O}}_{2}$ and, given this uncertainty, underlying mechanisms can only be speculated upon.

The reduction in peak HR might involve mechanisms involved in limiting maximum pacemaker rate, but we think this is unlikely and perhaps better reflects the reduced peak workload and other mechanisms which limit it (see below). Any reduction in peak SV might be linked to reduced cardiac contractility, greater afterload (high MAP), and/or lower preload linked to reduced muscle blood flow, venous return, and/or ventricular filling. Some of these effects might be linked to the lower circulating blood volume in DM, but more evidence is required to substantiate this effect on blood volume. The lower pulmonary [a-vO_2_] presumably reflects a greater mixed-venous [O_2_] and lower extraction of O_2_ in skeletal muscle. Impaired vascular control in skeletal muscle might underpin many of these effects, as outlined below in a more integrated interpretation of effects of DM on peak* and* dynamic responses of pulmonary $\dot{\text{V}}{\text{O}}_{2}$.

Compared with the effect of DM on peak $\dot{\text{V}}{\text{O}}_{2}$, there is a larger effect of DM on $\dot{\text{V}}{\text{O}}_{2}\tau$ in middle-aged individuals but no effect is observed in older subjects (Table 1). This effect is associated with a greater* increase* in pulmonary [a-vO_2_] relative to $\dot{\text{V}}{\text{O}}_{2}$ and CO which, in turn, reflects a lower mixed-venous [O_2_] and points to greater degree of perfusion-limited O_2_ exchange in contracting muscles [3]. Human studies and experimental models of prediabetes and diabetes provide strong evidence of impaired vasodilation in contracting skeletal muscle that is targeted at the arteriolar network. As mitochondrial consumption of O_2_ begins to rise in contracting myocytes, slowing of the vasodilation and increase in blood flow means that O_2_ flux between capillary blood and these myocytes becomes more “flow-limited” [3], capillary PO_2_ will fall to a greater extent [52], and mixed-venous [O_2_] will be lower. Under such conditions the control of the rise in muscle and pulmonary $\dot{\text{V}}{\text{O}}_{2}$ depends more heavily on perfusive (“O_2_ delivery”) as opposed to diffusive (“O_2_ utilisation”) processes and helps explain how vascular impairment might increase pulmonary [a-vO_2_] and $\dot{\text{V}}{\text{O}}_{2}\tau$ during submaximal exercise in DM.

How might these diabetes-induced changes in dynamic responses during submaximal exercise relate to impaired peak responses during maximal graded exercise? One possibility relates to the powerful and reversible effects of perfusion on muscle contractility [53–55] at moderate and higher workloads. Acute reductions in muscle perfusion induced by body tilting lead quickly to increased muscle fatigability during* submaximal* contractions at forces above 30% MVC [33, 56]. Similar manoeuvres during knee extensor exercise and cycle ergometer exercise slow the rise in muscle blood flow and pulmonary $\dot{\text{V}}{\text{O}}_{2}$ [57, 58] and, during cycling, increase electromyographic activity in thigh and leg muscles at high submaximal intensities and reduce peak heart rate, power output, and $\dot{\text{V}}{\text{O}}_{2}$ by 10–20% [58–60]. Thus, slowed dynamic responses of pulmonary $\dot{\text{V}}{\text{O}}_{2}$ observed in DM during more intense submaximal exercise (>80% VT) [18, 19] linked to increased vascular constraint in contracting muscles might reduce muscle contractility, necessitate an increased motor drive (central command) to maintain the required power output, and ultimately diminish the peak workload and heart rate achieved during a maximum graded test. In this way the slowed dynamic responses of muscle blood flow and $\dot{\text{V}}{\text{O}}_{2}$ during submaximal exercise contribute to the reduced peak HR, $\dot{\text{V}}{\text{O}}_{2}$, and exercise tolerance. The paradoxical blunting of peak [a-vO_2_] might reflect an altered balance of vascular resistances and blood flows in contracting (lower flow, lower venous O_2_) and noncontracting tissue (higher flow, higher venous O_2_) in the presence of an elevated mean arterial pressure and/or a reduced mass of contracting muscle.

The DM-induced increase in peak MAP is already manifest at lower workloads and appears very early during exercise at these workloads. The magnitude of this effect appears to be blunted in older individuals [11], and understanding this differential effect of age on the pressor response might be important to understanding whether exertional hypertension in DM reflects altered cardiovascular control or is simply a function of increased arterial stiffness. Increased central command and afferent input from skeletal muscles and peripheral chemoreceptors during submaximal exercise are thought to increase the operating point for blood pressure during graded exercise [6]. Whether or not such input is increased and contributes to exertional hypertension in DM is not known, but the coincidental timing of the blunted muscle hyperaemic response and increased blood pressure during the initial 30 s of exercise raises the possibility that impaired vascular control in muscle alters neural input to brainstem areas involved in cardiovascular control. It seems equally possible that the increased pressor response is due to a rapid increase in arterial inflow (i.e., CO) into a stiffer arterial system during the early period of exercise. Further investigation is required to clarify these possibilities.

## 9. Future Directions

Alterations to the dynamic response of hyperaemia in contracting muscle might be a central mechanism underlying the effects of DM on dynamic and peak responses of pulmonary $\dot{\text{V}}{\text{O}}_{2}$, exercise tolerance, and exertional hypertension during whole-body exercise. However, there is a paucity of data about dynamic responses of muscle hyperaemia during whole-body exercise and mechanisms underlying the impaired response observed during single-limb exercise. Although there is evidence of “endothelial dysfunction” in resting limbs in DM, this should not imply that it affects the control of muscle hyperaemia during exercise given the greater complexity and multiplicity of mechanisms involved. Further research is required to understand how DM impairs vascular control in contracting human muscle. Recent evidence of increased muscle sympathetic nerve activity in subjects with metabolic syndrome [61] and blunting of rapid vasodilation during calf muscle contractions in healthy subjects by activation of this response [62] suggests just one line of investigation into DM and vascular control in human skeletal muscle.

With respect to whole-body exercise, there is a lack of data about regional blood flows (in addition to contracting skeletal muscle) required to better understand how DM alters pulmonary $\dot{\text{V}}{\text{O}}_{2}$ and its Fick determinants. Assessment of dynamic responses of cardiac output, muscle blood flow, and other regional blood flows during whole-body exercise is technically challenging, and there is a need to improve the accuracy and temporal resolution of the required techniques to reveal more subtle aspects of these responses identified during single-limb exercise. Overcoming these technical limitations should help resolve underlying causes of exertional hypertension and clarify the relative influences of arterial stiffness and altered cardiovascular control. Simpler experiments might focus on clarifying the time-course of arterial pressure and cardiac output combined with assessment of the pressure waveform to establish the “dynamics” of the hypertensive response and identify the contribution of arterial stiffness to it. Finally, these cardiovascular consequences of DM need effective treatments given the cardiovascular risk and problem of physical inactivity associated with diabetes and obesity. Such treatments include exercise training, diet, and pharmacotherapy, and to our knowledge the effect of such treatments on cardiovascular control during exercise has received little attention in the literature and represents a fertile area for future research.

## Figures and Tables

**Table 1:** Data from multiple studies pertaining to the effects of type 2 diabetes mellitus on peak heart rate (HR), peak oxygen uptake ($\dot{\text{V}}{\text{O}}_{2}$), and the time constant of oxygen uptake ($\dot{\text{V}}{\text{O}}_{2}\tau$). Mean values are presented and the “Mean” corresponds to the average of the mean values from each study. “%diff” is the percentage difference between control and diabetic subjects. C = control subjects. DM = type 2 diabetes mellitus. “Mode” of exercise testing is cycling (C) or treadmill (T).

Reference	Sample size		Age (Y)			BMI			Peak HR (bpm)			Peak $\dot{\text{V}}{\text{O}}_{2}$ (L/min)			$\dot{\text{V}}{\text{O}}_{2}\tau$ (s)			Mode
	C	DM	C	DM	%diff	C	DM	%diff	C	DM	%diff	C	DM	%diff	C	DM	%diff	
Adult female, untrained, <60 y																		
[17]	21	15	30–55	30–55		29.1	30.9		163	163	0.0	1.77	1.37	−22.6	31.5	37.1	17.8	C
[12]	10	10	39.3	42.5	8.1	28.3	31.9	12.7	166	169	1.8	1.64	1.52	−7.3	—	—	—	C
[18]	10	10	37	42	13.5	30.8	33.1	7.5	168	167	−0.6	1.73	1.36	−21.4	27.8	36.8	32.4	C
[19]	9	9	42.5	49.1	15.5	29	34.4	18.6	165	145	−12.1	1.77	1.4	−20.9	24.8	39.1	57.7	C
[20]	16	16	53.4	54.8	2.6	29	30.2	4.1	154	154	0.0	1.6	1.5	−6.3	33.6	43.3	28.9	C
Mean	**12.7**	**11.3**	**37.5**	**40.7**	**7.6**	**29.6**	**33.1**	**13.2**	**167.0**	**161.7**	−**3.1**	**1.76**	**1.56**	−**12.2**	**29.4**	**39.1**	**34.2**	

Adult male, untrained, <60 y																		
[17]	13	14	30–55	30–55		28.5	30.6	7.4	161	163	1.2	2.46	2.26	−8.1	34.8	45.1	29.6	C
[20]	16	16	57.8	57.4	−0.7	28.2	29	2.8	161	157	−2.5	2.8	2.5	−10.7	35.3	43.8	24.1	C
[11]	11	15	48	52	8.3	28.8	29.3	1.7	169	160	−5.3	3.2	2.5	−21.9	26.8	41.6	55.2	C
[21]	11	8	49	53	8.2	27.5	28.7	4.4	175	169	−3.4	2.83	2.31	−18.4	—	—	—	C
Mean	**12.8**	**13.3**	**51.6**	**54.1**	**5.3**	**28.3**	**29.4**	**4.1**	**166.5**	**162.3**	−**2.5**	**2.8**	**2.4**	−**14.8**	**32.3**	**43.5**	**36.3**	

Mixed (4 F/6 M), untrained, <60 y																		
[22]	10	10	51	50	−2.0	27	30	11.1	161	153	−5.0	2.3	1.9	−17.4	—	—	—	T

Adult male, untrained, >60 y																		
[23]	12	12	62	65	4.8	28.4	29.2	2.8	—	—		2.72	1.98	−27.2	41	43	4.9	C
[11]	10	18	64	64	0.0	28.2	30.3	7.4	156	154	−1.3	2.7	2.4	−11.1	40.5	41.1	1.5	C

Adult female, untrained (UT) and trained (T), <60 y																		
[24]	9 (UT)	8 (UT)	37	43	16.2	30.3	30.9	2.0	167	166	−0.6	1.74	1.42	−18.4	27.7	47.7	72.2	C
	9 (T)	8 (T)	37	43	16.2	31.8	30.5	−4.1	164	164	0.0	1.86	1.75	−5.9	18.9	37.4	97.9	C

Adolescents																		
[25]	10	8	15.2	14.9	−2.0	31.1	38.3	23.2	186	172	−7.5	2.07	2.18	5.3	—	—	—	C
[14]	27	13	15.1	15.4	2.0	35.5	36.5	2.8	175.7	174.2	−0.9	2.2	2.12	−3.6	—	—	—	C
